# Metabolic engineering of the pentose phosphate pathway for enhanced limonene production in the cyanobacterium ***Synechocysti****s* sp. PCC 6803

**DOI:** 10.1038/s41598-017-17831-y

**Published:** 2017-12-13

**Authors:** Po-Cheng Lin, Rajib Saha, Fuzhong Zhang, Himadri B. Pakrasi

**Affiliations:** 1 Department of Energy, Environmental & Chemical Engineering, Washington University, St. Louis, MO 63130 USA; 2 Department of Biology, Washington University, St. Louis, MO 63130 USA; 3 Present Address: Department of Chemical & Biomolecular Engineering, University of Nebraska–Lincoln, Lincoln, NE 68588 USA

## Abstract

Isoprenoids are diverse natural compounds, which have various applications as pharmaceuticals, fragrances, and solvents. The low yield of isoprenoids in plants makes them difficult for cost-effective production, and chemical synthesis of complex isoprenoids is impractical. Microbial production of isoprenoids has been considered as a promising approach to increase the yield. In this study, we engineered the model cyanobacterium *Synechocystis* sp. PCC 6803 for sustainable production of a commercially valuable isoprenoid, limonene. *Limonene synthases* from the plants *Mentha spicata* and *Citrus limon* were expressed in cyanobacteria for limonene production. Production of limonene was two-fold higher with limonene synthase from *M*. *spicata* than that from *C*. *limon*. To enhance isoprenoid production, computational strain design was conducted by applying the OptForce strain design algorithm on *Synechocystis* 6803. Based on the metabolic interventions suggested by this algorithm, genes (*ribose 5*-*phosphate isomerase* and *ribulose 5*-*phosphate 3*-*epimerase*) in the pentose phosphate pathway were overexpressed, and a *geranyl diphosphate synthase* from the plant *Abies grandis* was expressed to optimize the limonene biosynthetic pathway. The optimized strain produced 6.7 mg/L of limonene, a 2.3-fold improvement in productivity. Thus, this study presents a feasible strategy to engineer cyanobacteria for photosynthetic production of isoprenoids.

## Introduction

Recent studies have demonstrated the potential of using cyanobacteria as biological platforms to produce fuels and high-value chemicals^1,2^. Harnessing solar energy using the photosynthetic apparatus, atmospheric CO_2_ is fixed into sugars, which can be further converted to desired products by engineered cyanobacteria. Due to the recent development of genetic tools for model cyanobacteria^3^, expression of heterologous genes and pathways has become more feasible, thus facilitating the construction of engineered cyanobacteria for biotechnological applications. In this study, we engineered the model cyanobacterium *Synechocystis* sp. PCC 6803 (hereafter, *Synechocystis* 6803) for production of a commercially valuable isoprenoid, limonene.

Isoprenoids are one of the most diverse groups of natural products, with more than 55,000 compounds^4^. Isoprenoids have multiple commercial applications, including natural pharmaceuticals, nutraceuticals, solvents, and perfume components^5,6^. To date, commercially-used isoprenoids are mainly extracted from plants, but the low quantities of these naturally-produced chemicals have become an impediment for cost-effective production. Successful microbial production of valuable isoprenoids by engineered yeast and *E*. *coli* have been demonstrated^7,8^, whereas fewer researchers have studied production of isoprenoids by cyanobacteria. To improve photosynthetic production of isoprenoids, optimization of isoprenoid biosynthetic pathways in cyanobacteria is needed using metabolic engineering coupled with computational approaches.

Limonene is a 10-carbon isoprenoid produced by plants. (R)-limonene has a characteristic fragrance of orange, and commonly exists in the rinds of citrus fruits. It is commercially used as a fragrance in perfumes or a solvent in cleaning products. (S)-Limonene is a precursor for the biosynthesis of (S)-menthol, which is the major component of mint. Recently, limonene has been evaluated as a “drop-in” replacement for diesel^9^ and jet fuels^10^. The fully hydrogenated form of limonene was used as a diesel additive, exhibiting similar chemical properties compared to diesel fuel^9^. Moreover, the physical properties of limonene, such as viscosity, freezing point, and boiling point, are highly comparable to aviation fuel Jet A-1^10^.

Cyanobacteria use the methylerythritol 4-phosphate (MEP) pathway to produce isopentenyl pyrophosphate (IPP) and dimethylallyl pyrophosphate (DMAPP), which are the building blocks for isoprenoid biosynthesis. The MEP pathway is a seven-step pathway that starts with glyceraldehyde 3-phosphate (GAP) and pyruvate, and ends with IPP and DMAPP. Further, IPP and DMAPP undergo a series of head-to-tail condensations to produce diphosphate substrates, which are then converted to isoprenoids by isoprenoid synthases. To increase isoprenoid production, the amounts of IPP and DMAPP need to be enhanced by increasing the carbon flux toward the MEP pathway.

Attempts have been made to engineer the MEP pathway for improving cyanobacterial limonene production. However, production titers are extremely low compared to other compounds such as ethanol^11^, butanol^12^, and free fatty acid^13^. Genes involving in the bottlenecks of the MEP pathway were overexpressed in *Synechocystis* 6803^14^. The recombinant strain showed a 1.4-fold increase of limonene, and the final titer reached 1 mg/L after 30-day cultivation^14^. In addition, researchers used similar strategies to engineer the MEP pathway in the nitrogen-fixing cyanobacterium *Anabaena* sp. PCC 7120 for production of limonene^15^. The limonene yield increased up to 6.8-fold. However, the final titer remained low (0.5 mg/L over 12-day incubation)^15^.

A previous *in vitro* study suggested that isoprenoid production in *Synechocystis* 6803 is stimulated by compounds in the pentose phosphate (PP) pathway but not by substrates in the MEP pathway^16^. Using *Synechocystis* 6803 cell extracts, isoprenoid biosynthesis was significantly improved by supplying xylulose 5-phosphate (X5P) in the PP pathway, whereas providing substrates (GAP, pyruvate, and MEP) in the MEP pathway showed lower stimulation of isoprenoid production^16^. These results indicated a connection between the PP pathway and isoprenoid production in *Synechocystis* 6803.

In addition to experimental engineering approaches, computational strain design techniques can be useful to develop non-intuitive genetic interventions to achieve the desired level of production of a particular bioproduct. To this end, the OptForce procedure^17^ first characterizes the wild-type strain in the form of reaction flux ranges by utilizing the ^13^C MFA (**M**etabolic **F**lux **A**nalysis) flux estimations as additional regulations. OptForce then contrasts the wild-type flux ranges with those in the overproducing phenotype. As a result, the algorithm identifies a set of genetic interventions (i.e., up/down-regulations and deletions) that must happen in the metabolic reaction network for a desired level of yield. Finally, OptForce pinpoints the minimal interventions (from these changes) that are directly related to achieving the desired yield. These strategies can then be tested in an experimental setting.

**Figure 1:**
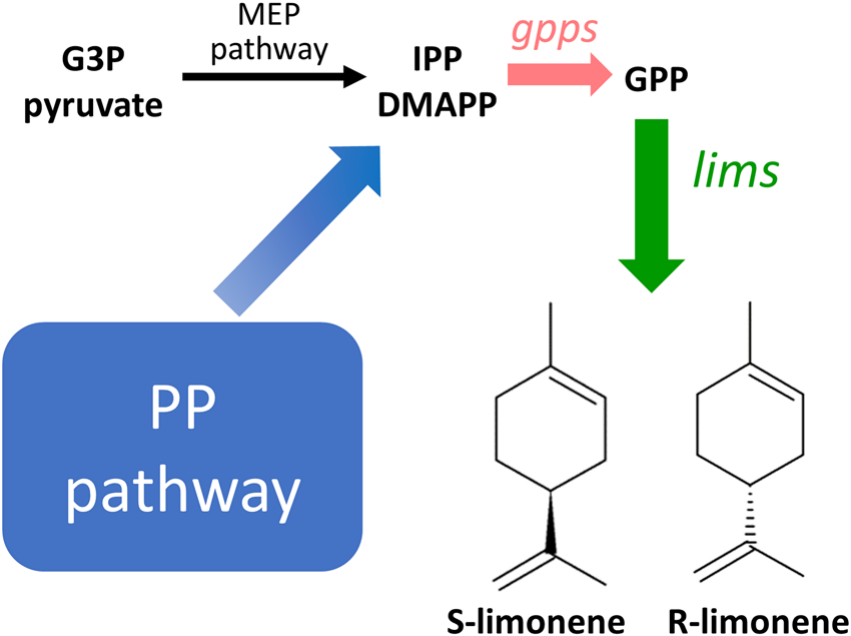
Schematic representation of engineering *Synechocystis* 6803 for production of limonene. Codon-optimized *limonene synthases* from *Mentha spicata* and *Citrus limon* were heterologously expressed in *Synechocystis* 6803 to produce S-limonene and R-limonene, respectively. The limonene biosynthetic pathway was optimized by overexpressing genes in the pentose phosphate (PP) pathway and a *geranyl diphosphate synthase* from *Abies grandis*. G3P, glyceraldehyde 3-phosphate; MEP, methylerythritol-4-phosphate; IPP, isopentenyl diphosphate; DMAPP, dimethylallyl diphosphate; GPP, geranyl diphosphate; *lims*, limonene synthase; *gpps*, geranyl diphosphate synthase.

In this work, we engineered *Synechocystis* 6803 for photosynthetic limonene production (Fig. 1). To construct limonene-producing strains, genes encoding *limonene synthase* (*lims*) from *Mentha spicata* and *Citrus limon* were introduced into *Synechocystis* 6803. For generating computation-driven non-intuitive strain engineering strategies, we applied the OptForce algorithm^17^ on the genome-scale *Synechocystis* 6803 model *i*Syn731^18^ and also utilized ^13^C MFA flux estimations^19^ under photosynthetic wild-type condition. OptForce predicted the up-regulation of two PP pathway genes, *ribose 5*-*phosphate isomerase* (*rpi*) and *ribulose 5*-*phosphate 3*-*epimerase* (*rpe*), in limonene-producing strains in order to divert the carbon flux toward limonene production. Furthermore, based on the prediction made by OptForce to further improve limonene production, a *geranyl diphosphate synthase* (*gpps*) from *Abies grandis* was expressed to optimize the limonene production pathway. The final recombinant strain led to a 2.3-fold improvement in yield, producing 6.7 mg/L of limonene in 7 days. The metabolic engineering strategies used in this study demonstrate the feasibility of increasing limonene production in *Synechocystis* 6803 and can be applied to phototrophic production of other high-value isoprenoids.

## Results

### **Engineering*Synechocystis*6803 for production of limonene**

**Figure 2:**
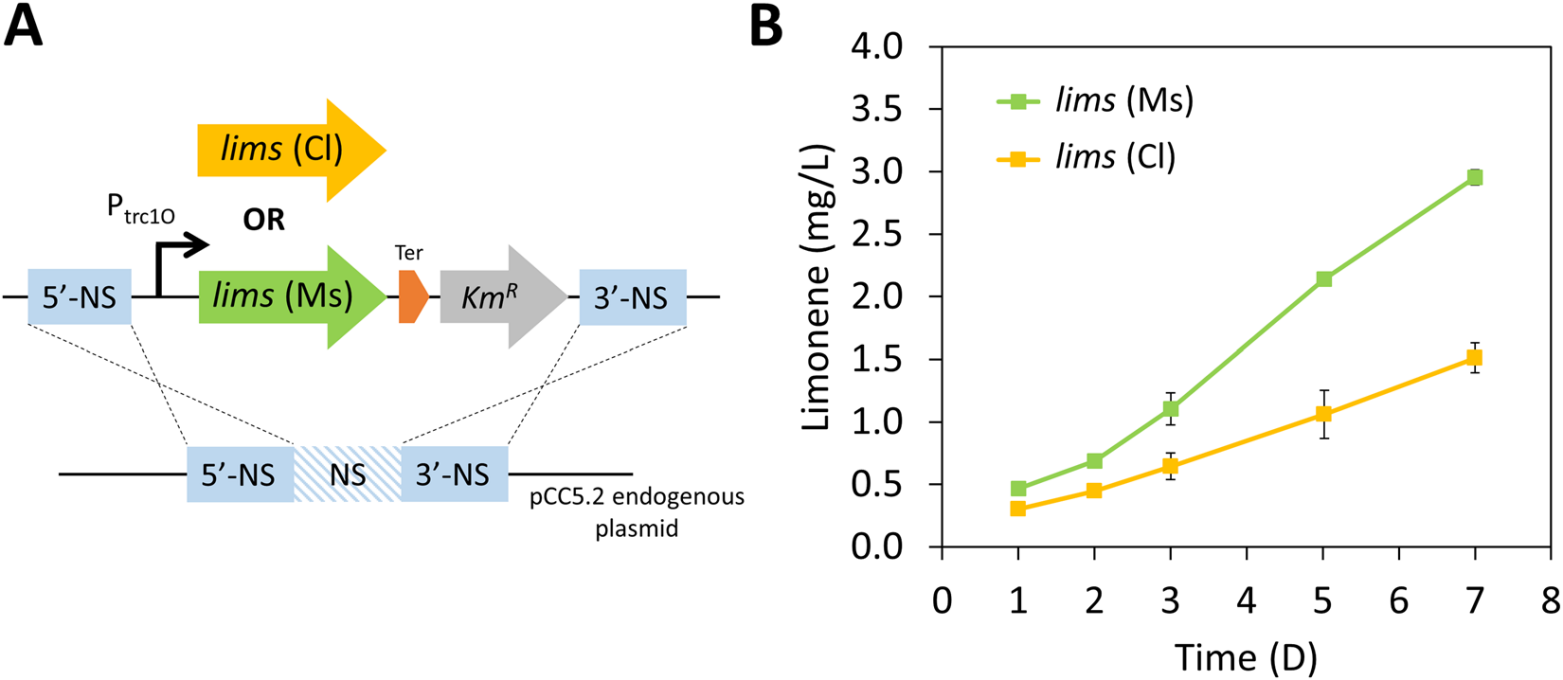
Production of limonene by engineered *Synechocystis* 6803. (**A**) Introduction of *limonene synthases* into a neutral site on endogenous pCC5.2 plasmid to create limonene-producing mutants. (**B**) Time-course limonene production. Results were mean  $\pm$ SD of three biological replicates. *lims* (Ms), *limonene synthase* from *M*. *spicata*; *lims* (Cl), *limonene synthase* from *C*. *limon*; P_trc1O_, trc1O promoter; NS, neutral site; Ter, terminator; Km^R^, kanamycin resistance cassette.

Limonene is a C10 cyclic isoprenoid converted from geranyl diphosphate (GPP). Due to the complex nature of carbocation rearrangement from GPP to limonene, limonene synthase produces not only limonene but also other monoterpenes such as bicyclic α-pinene and acyclic mycene^20^. To avoid the production of other unwanted byproducts, we chose limonene synthases which have the highest specificity for limonene production. Based on previous studies, limonene synthase from *Citrus limon* and *Mentha spicata* produce limonene of high purity. Expression of each of these limonene synthases in *E*. *coli* showed that the former produces 99% pure (R)-limonene^21^, and the latter generates 94% of (S)-limonene^22^. The coding sequences of *lims* were codon optimized for *Synechocystis* 6803, and the plastid targeting sequences were removed^23,24^. The truncated enzyme is known to have better catalytic activity than the native protein^25^. Genes were cloned into a pCC5.2 neutral-site-targeting plasmid and driven by the trc1O promoter for higher level expression of *lims* (Fig. 2A). Expression of an enhanced yellow fluorescent protein (EYFP) from the pCC5.2 endogenous plasmid is 8 to 14 times higher than that on the chromosome^26^.

When the *lims* was cloned into a suicide plasmid and transformed into *E*. *coli*, we found that the gene accumulated random mutations in the *E*. *coli* host, leading to changes in amino acid residues or truncated proteins. This was presumably because the *lims* product is toxic to *E*. *coli* cells. To introduce a *lims* without mutations into *Synechocystis* 6803, we circumvented the *E*. *coli* cloning step by first cloning the *lims* into the suicide plasmid via Gibson assembly, and used the assembled product as template for PCR to amplify the *lims* cassette flanked by upstream and downstream homologous sequences of the neutral site in pCC5.2^26^. Subsequently, the PCR product was directly used for natural transformation into *Synechocystis* 6803. The *lims* was introduced into *Synechocystis* 6803 genome via double homologous recombination (Fig. 2A). DNA sequencing results showed that the *lims* has no mutation in *Synechocystis* 6803 (data not shown). Mutants were fully segregated after re-streaking the cells several times on BG-11 plates with antibiotics.

Limonene production by engineered *Synechocystis* 6803 was tested by incubating cultures for 7 days. Because of the volatility of limonene, a dodecane overlay was applied on cultures to collect limonene in the organic layer. It has been reported that over 99% of limonene escapes from the cyanobacterial cultures^14^, and covering an organic overlay on cultures had little influence on growth in cyanobacteria^23^. The limonene yield by the strain expressing *lims* from *M*. *spicata* was two-fold higher than that by the strain expressing *lims* from *C*. *limon* (Fig. 2B). These results suggest that the limonene synthase from *M*. *spicata* exhibited better catalytic activity in *Synechocystis* 6803, and hence, the strain was used for further engineering.

### Computational modeling

**Figure 3:**
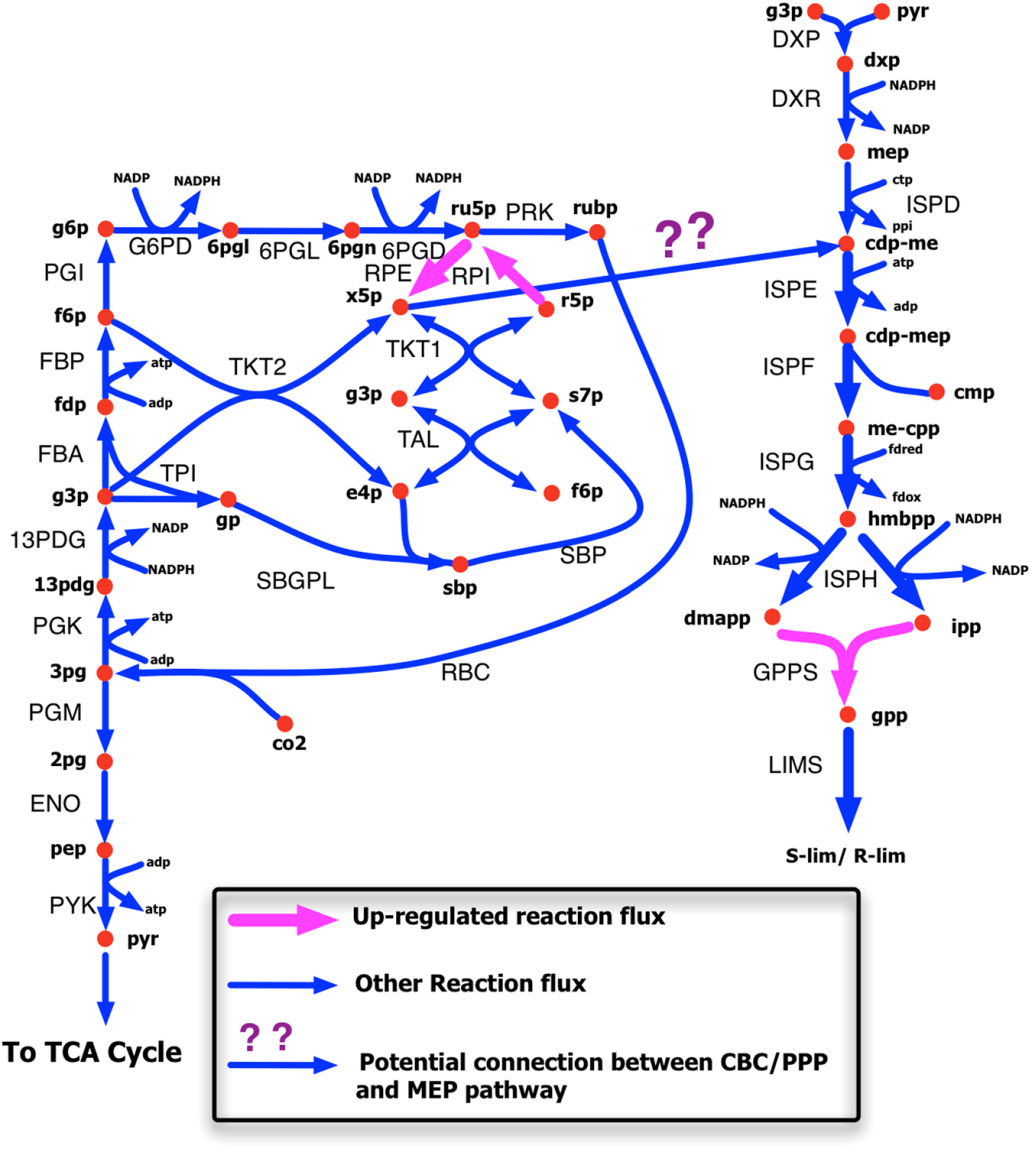
Metabolic interventions predicted by the OptForce algorithm. Up-regulation of *rpe*, *rpi*, and *gpps* (showed with pink arrows) leads to the improved production yield of limonene.

The *i*Syn731 metabolic model of *Synechocystis* 6803^18^ was used to perform the computational strain designs using the OptForce algorithm^17^ for overproduction of limonene. Based on the current understanding as reported in literature^16,27^, a connection between Calvin Benson Cycle (CBC)/PP pathway and MEP pathway (Fig. 3) was included in the *i*Syn731 model. By superimposing the photoautotrophic flux measurements^19^ of 31 reactions of central carbon metabolism including the CBC and PP pathways of *Synechocystis* 6803 onto the *i*Syn731 model, the phenotypic space of the base strain was defined. All simulations were performed for a basis of 100 millimoles of CO_2_ plus H_2_CO_3_ uptake and unlimited photon supply^19^. The uptake fluxes for the remaining metabolites present in the BG11 medium was set to -1,000 and the non-growth associated ATP maintenance was set at 8.39 mmole/gDW-h. In addition, the biomass flux was fixed at the optimal value subject to the experimental flux measurements^19^. The upper bound of the fluxes of the remaining reactions was set to 1,000 mmole/gDW-h, whereas the lower bound was set to zero and -1,000 mmole/gDW-h for irreversible and reversible reactions, respectively.

Similarly, the limonene overproducing phenotype was obtained by maximizing and minimizing each flux of the metabolic model iteratively subject to the network stoichiometry, uptake and medium conditions, regulatory constraints, and overproduction target. In this work, a minimum production yield of 85% of the theoretical maximum of limonene (*i*.*e*., 15.3 mmole/gDW-h) was set as the overproduction target, while the biomass flux was constrained to be at least 10% of its theoretical maximum (i.e., 0.021 h^−1^) with the basis of 100 millimoles of carbon fixed (i.e., CO_2_ plus H_2_CO_3_). The remaining parameter values including medium conditions and regulatory constraints were the same as those in the wild-type. By contrasting the maximal range of flux variability between the wild-type strain and the over-producing strain to meet the pre-specified yield of limonene, OptForce was used to identify the minimal set of genetic interventions (i.e., deletions and up-/down-regulations). In order to first explore non-intuitive interventions, reactions from the MEP and isoprenoid biosynthesis pathways were not considered as the candidates for any form of intervention. Integer cuts were used to identify alternative optimal solutions (i.e., alternative genetic intervention choices) to achieve the minimum production yield of limonene as specified earlier. The termination criterion for the OptForce procedure was set as either meeting a production yield of at least 85% of the theoretical maximum for limonene or exceeding the maximum allowable number of reaction interventions (i.e., three). Note that the OptForce procedure works at the reaction level, which is why the set of genetic manipulations can subsequently be identified by using gene-protein-reaction (GPR) associations from the *i*Syn731 model. Thus, the OptForce procedure identified up-regulation of *rpi* and *rpe* as the best possible solution, which can lead up to limonene yield at 89% of its theoretical maximum (i.e., 16.02 mmole/gDW-h). By up-regulating these two genes, OptForce suggested to force more flux from the CBC/PP pathway toward MEP pathway that can ultimately increase the production yield of limonene (Fig. 3). Once the set of non-intuitive interventions was obtained, as a next step, it was logical to explore if their combination with any of the intuitive one(s) from the MEP and isoprenoid biosynthesis pathways could further improve the limonene production yield that was otherwise not possible to achieve individually (*i*.*e*., by the non-intuitive candidates or by the intuitive ones). With a target of a minimum production yield of 90% of the theoretical maximum of limonene, the OptForce procedure identified the up-regulation of *gpps*, *rpe*, and *rpi* that could lead the limonene production yield to 16.56 mmole/gDW.h (*i*.*e*., 92% of its theoretical maximum). Thus, the proposed interventions combined the amplification (*i*.*e*., push) of flux from the CBB/PP pathway to MEP pathway with a similar increase (*i*.*e*., pull) in the flux of the limonene synthesis. As reported in the literature^28^, this kind of push-and-pull strategy can achieve the desired level of production yield with minimal effects caused by feedback inhibition.

### Genetic interventions of the PP pathway to improve limonene production

**Figure 4:**
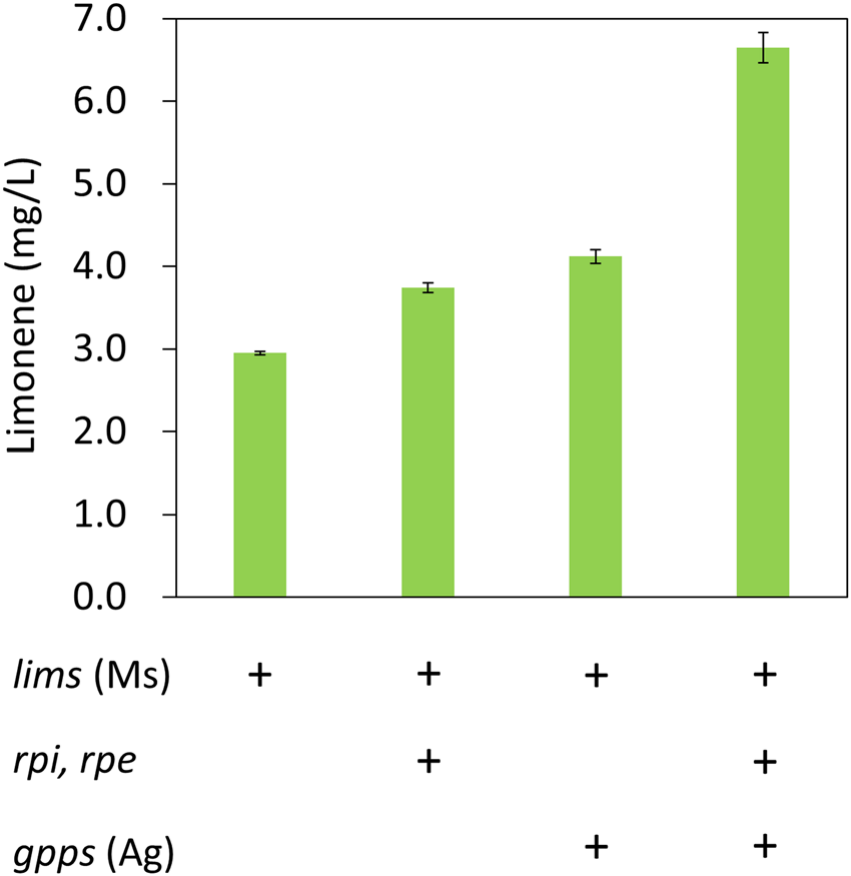
Increased limonene production by genetic modifications. Two genes in the PP pathway (*rpi* and *rpe*) were overexpressed in *Synechocystis* 6803 to divert carbon flux toward limonene production. The *Abies grandis* GPPS 2 that specifically produce GPP was expressed to ensure sufficient GPP for limonene production. Ms, *Mentha spicata*; Ag, *Abies grandis*. Results were mean  $\pm$ SD of three biological replicates.

Based on the prediction of the OptForce procedure, up-regulation of *rpi* and *rpe* genes in the PP pathway increases the flux toward limonene production. To test this hypothesis, the *rpi* and *rpe* genes driven by the *Synechocystis* 6803 native *rbcL* promoter were expressed on a replicating plasmid in the limonene-producing strain, resulting in 1.3-fold increase in limonene yield (3.7 mg/L) after 7 days of cultivation (Fig. 4). Furthermore, we introduced a gene encoding a specific GPP synthase (GPPS) to optimize the limonene biosynthetic pathway. In *Synechocystis* 6803, formation of GPP is catalyzed by a farnesyl diphosphate (FPP) synthase, CrtE. It performs consecutive condensation of IPP with DMAPP, and only synthesizes GPP as an intermediate^29^. Although the PP pathway was engineered to stimulate the limonene yield, it is possible that the native isoprenoids pathway in *Synechocystis* 6803 provides insufficient GPP for limonene production since the flux is diverted toward FPP formation for pigment synthesis. In addition, OptForce also predicted an increase (i.e., from 89% to 92% of maximum theoretical limonene yield) when up-regulation of *rpe* and *rpi* was combined with the up-regulation of *gpps*. It was reported that the GPPS 2 from *Abies grandis* specifically produces GPP^30^. Expressing this specific *gpps* with *lims*, the limonene yield increased 1.4-fold (4.1 mg/L) (Fig. 4). Finally, coexpression of *rpi*, *rpe*, *gpps* and *lims* resulted in a remarkable (2.3-fold) enhancement in productivity (6.7 mg/L) (Fig. 4).

### **Pigment content in engineered*Synechocystis*6803**

**Figure 5:**
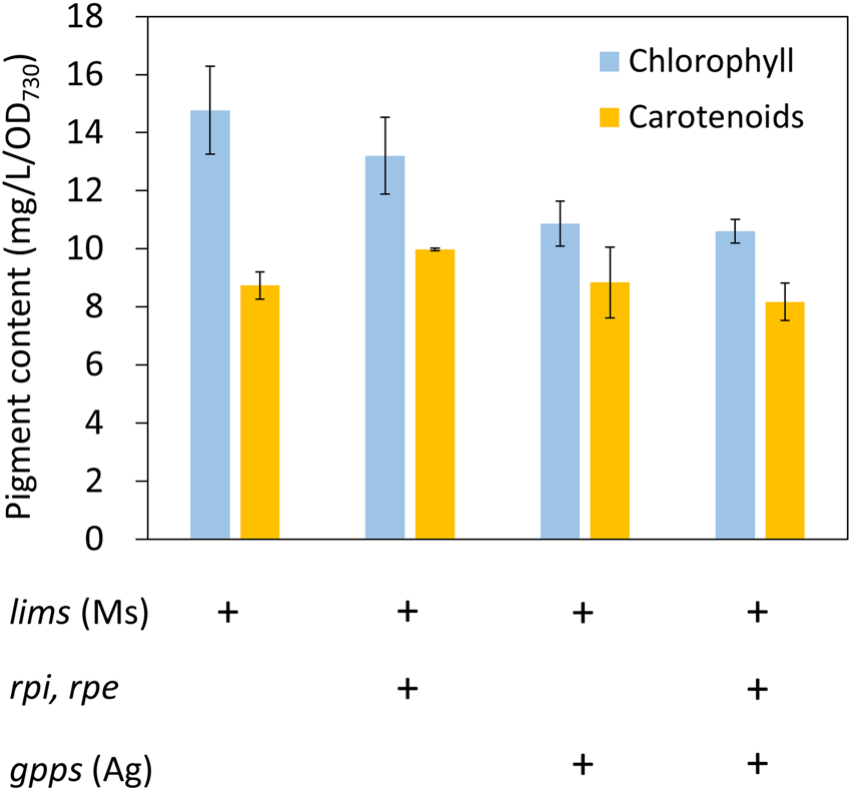
Pigment content of engineered *Synechocystis* 6803 strains. Chlorophyll and carotenoid contents of limonene-producing strains. Ms, *Mentha spicata*; Ag, *Abies grandis*. Results were mean ± SD of three biological replicates.

Carotenoids and the phytol tail of chlorophyll, photosynthetic pigments, are derived from geranylgeranyl diphosphate (GGPP), a C20-intermediate for isoprenoid synthesis. Hence, production of limonene is expected to divert carbon flux away from pigment synthesis. To investigate the effect of limonene production on pigment content in engineered *Synechocystis* 6803, we extracted and quantified the chlorophyll and carotenoid contents. The chlorophyll content decreased over 30% in the *gpps* expression strains, whereas carotenoid levels were similar among the limonene-producing strains (Fig. 5). These results indicate that the specific GPPS diverts the carbon flux away from pigment synthesis.

## Discussion

In this study, we combined metabolic engineering with model-driven strain design strategies to engineer *Synechocystis* 6803 for enhanced limonene production. To generate limonene-producing *Synechocystis* 6803, we first constructed a suicide plasmid^26^ to engineer the *lims* into the neutral site on the pCC5.2 endogenous plasmid via double homologous recombination. This is the first time that the endogenous plasmid of *Synechocystis* 6803 has been used for enhanced production for the purpose of metabolic engineering. Expression of a gene on the pCC5.2 plasmid leads to higher expression level than that on the chromosome as well as the RSF1010 self-replicating plasmid^26^. Furthermore, during the stationary phase of cell growth, the copy numbers of the endogenous plasmids (pCA2.4, pCB2.4, pCC5.2) in *Synechocystis* 6803 are 3 to 7 per chromosome^31^. Using the endogenous plasmid to express the *lims* gene driven by the constitutive promoter trc1O allows high expression level at the stationary phase, decoupling growth and production, and thus leading to higher levels of production of limonene.

The higher yield with limonene synthase from *M*. *spicata* than that from *C*. *limon* may be due to the difference in enzyme kinetics of LIMS. Unfortunately, the kinetic parameters (both *K*_m_ and *k*_cat_) are only available for the enzyme from *M*. *spicata* ^25^. In addition, it may be attributed to different protein expression levels. Although the same promoter was used to control the *lims* from two plant species, protein expression may vary because of different mRNA sequences and codon usage. To date, the highest reported limonene productivity in cyanobacteria was achieved by engineered *Synechococcus* sp. PCC 7002^23^. In their study, only a *lims* from *M*. *spicata* was overexpressed, and the yield was over 4 mg/L in 4 days^23^. Our results also suggested that the LIMS from *M*. *spicata* performed better in limonene production (Fig. 2B). The doubling time of *Synechococcus* 7002 is shorter than *Synechocystis* 6803^32^. Thus, the higher limonene yield from *Synechococcus* 7002 may be due to its faster growth rate. A recent study engineered *Synechococcus elongatus* PCC 7942 to produce limonene, achieving a 100-fold improvement in productivity^33^. However, it should be noted that such significant increase is due to the low productivity of the original strain, which produced merely 8.5 μg/L/OD/d of limonene. The best producing strain in this study, with a *lims* (*M*. *spicata*) controlled by the pea plant *psbA* promoter, produced 2.5 mg/L limonene in 5 days^33^.

Previously, researchers have engineered *Synechocystis* 6803 for limonene production by overexpressing genes involved in the bottleneck steps of the MEP pathway^14^. It is known that enzymes 1-deoxy-D-xylulose-5-phosphate synthase (DXS) and isopentenyl diphosphate isomerase (IDI) catalyze the rate-limiting reactions in the MEP pathway^34,35^. With the introduction of an additional copy of endogenous *dxs*, *idi*, and *gpps* genes, the engineered *Synechocystis* 6803 produced 1.4-fold higher yield than that of the parent strain^14^. However, such improvement was less effective than the strategy used in the current study. As mentioned in the Results section, the endogenous *gpps* gene may not be suitable for enhancing the production of limonene. In addition, the MEP pathway is highly regulated at genetic and metabolic levels^36^. Expressing endogenous genes in the MEP pathway may be subject to native regulations, presenting a less effective engineering approach.

Instead of manipulating the MEP pathway, we took a systematic model-driven metabolic engineering approach for finding genetic interventions in order to increase the limonene production yield. As explained in the Materials and Methods section, the OptForce procedure finds the minimal interventions to reach a desired production target. To this end, we employed OptForce on our previously developed genome-scale model *i*Syn731 in order to ‘*push*’ more flux to MEP pathway and also to create better ‘*pull*’ for limonene synthesis (Fig. 3). From this *in silico* analysis, by up-regulating *rpe* and *rpi*, the metabolite pool of X5 P was found to be increased that, eventually, led to increased flux through the connection between the CBC/PP pathway and the MEP pathway. In addition, up-regulation of *gpps* created an improved ‘*pull*’ for limonene synthesis. Thus, the combination of this push-and-pull mechanism was proposed to be the best strategy to improve limonene yield by circumventing additional regulations (e.g. feedback inhibition). Interestingly, the same rationale could be applied to engineer cyanobacteria to produce other isoprenoid compounds.

Expression of the specific *gpps* modestly increased the limonene titer (Fig. 4), whereas the cellular chlorophyll content was greatly influenced (Fig. 5). Synthesis of limonene and the phytol tail of chlorophyll requires the same precursors, IPP and DMAPP. Table 1 compares the changes in chlorophyll and limonene contents between the strain with *lims* only and the two *gpps*-expressing strains. Compared to the *lims*-expressing strain, additional expression of *gpps* resulted in similar level of decrease in chlorophyll content in both strains. However, expression of *rpi* and *rpe* genes further led to 1.5-fold higher limonene productivity in the *gpps*-expressing strains (122 vs. 81 μM/OD_730_ isoprene unit). This result suggests that up-regulation of *rpi* and *rpe* enhances the carbon flux toward limonene synthesis.

**Table 1:** Comparison of chlorophyll and limonene contents.

Strains	lims	lims, gpps	lims, gpps, rpi, rpe
Chlorophyll*	66	49	47
Limonene*	53	81	122

*Presented as μM/OD_730_ isoprenes.

Our results showed that overexpressing the genes in the PP pathway led to improved limonene production, suggesting an unidentified connection between the PP pathway and isoprenoids biosynthesis (Fig. 4). Our observation is consistent with previous *in vitro* study using *Synechocystis* 6803 cell lysate^16^. However, the connection between the PP pathway and isoprenoids biosynthesis remains to be elucidated. It was first shown that *in vitro* isoprenoid production increased significantly by providing substrates in the PP pathway^16^, while a recent study showed that increased production of isoprenoids by PP pathway substrates does not occur through the MEP pathway^37^. By removing the terminal enzyme of the MEP pathway in *Synechocystis* 6803 cell lysate, isoprenoid synthesis still increased by substrates in the PP pathway^37^. Taken together, it is still unclear how the PP pathway and isoprenoid production are connected in *Synechocystis* 6803. While our results made a strong argument for this connection, further investigation needs to be conducted to explore the details in terms of chemical conversions and genes/enzymes associated. From the modeling context, these details sometimes do not make much of a difference if they only involve aggregating linear reaction steps.

## Conclusions

In this study, we engineered the model cyanobacterium *Synechocystis* 6803 to produce the isoprenoid, limonene. We applied computational strain design by using the OptForce procedure to identify minimal genetic interventions for improving limonene yield. Based on the prediction, the *rpi* and *rpe* genes in the PP pathway were overexpressed, and a specific *gpps* was introduced to optimize the limonene biosynthetic pathway. The final engineered strain produced 6.7 mg/L of limonene, which is a 2.3-fold improvement in productivity. The approach that we demonstrated can be applied to engineer cyanobacteria to produce other valuable isoprenoids.

## Materials and Methods

### Chemicals and reagents

All chemicals were purchased from Sigma-Aldrich (St. Louis, MO, USA) unless otherwise specified. Phusion DNA polymerase were purchased from Thermo Fisher Scientific (Waltham, MA, USA).

### Culture medium and condition

All strains were maintained in liquid BG-11 medium or on solid BG-11 plates with appropriate antibiotics at 30 °C continuous white light (50 μmoles photons m^−2^s^−1^).

### DNA manipulations

Coding sequences of *lims* from *Mentha spicata* and *Citrus limon* were codon optimized for *Synechocystis* 6803 and synthesized by IDT (San Jose, CA, USA). The genes were cloned into a suicide plasmid, allowing gene insertion into the neutral site (NSP1) on the endogenous plasmid pCC5.2 in *Synechocystis* 6803^26^. The constructed plasmids were directly used as templates for PCR to amplify a fragment which contains the *lims* and a kanamycin resistance cassette flanking by upstream and downstream homologous sequences of the NSP1 (Fig. 2A). The PCR product was then purified by DNA electrophoresis, and the linear DNA was transformed into *Synechocystis* 6803. The *rpi*, *rpe*, and *gpps* genes were cloned into a broad-host-range plasmid RSF1010 harboring a spectinomycin resistance cassette^38^. All the cloning works were done by Gibson isothermal DNA assembly method^39^.

### Strains construction and transformation

The *lims* expression cassette was transformed into *Synechocystis* 6803 through homologous recombination. Cells at mid-log phase (OD_730_ of 0.4 to 0.6) were incubated with 600 ng of linear DNA overnight at 30 °C in the dark. Cells were then grown on BG-11 plates supplemented with 10 μg/mL of kanamycin for selection of transformants. Colonies were patched on BG-11 plates with 20 μg/mL of kanamycin for segregation. PCR was used to verify strain segregation. For the construction of *rpi*, *rpe*, and *gpps* expressing strains, self-replicating plasmids (600 ng per transformation) were transformed into the strain expressing *lims*. Transformants were selected by BG-11 plates with 2 μg/mL of spectinomycin and 5 μg/mL of kanamycin.

### Limonene production by engineered cyanobacteria

Strains were inoculated in BG-11 medium with kanamycin (10 μg/mL) and spectinomycin (4 μg/mL) to mid-log phase at 30 °C with continuous white light (50 μmoles photons m^−2^s^−1^). Cells were collected by centrifugation at 7,000 x *g*, and washed by BG-11 medium to remove antibiotics. To test limonene production, the initial OD_730_ was adjusted to 0.34 (~0.5 g/L of biomass), and 50 mL of cell cultures were grown in 250-mL flasks at 30 °C with continuous white light (130 μmoles photons m^−2^s^−1^). A 10% (v/v) dodecane overlay was covered on top of cultures to trap evaporated limonene.

### Quantification of limonene

Limonene samples were prepared by diluting 10 μL of dodecane overlay in 990 μL of ethyl acetate, and analyzed using a gas chromatography instrument with a flame ionization detector (Hewlett-Packard model 7890 A, Agilent Technologies, CA, USA) equipped with a 30 m DB5-MS column (J&W Scientific). The oven temperature program initiated at 60 °C, and increased at 12 °C/min to 300 °C. Limonene was quantified using a (R)-limonene standard.

### Identification of engineering interventions via OptForce

We applied the OptForce algorithm^17^ on the genome-scale *Synechocystis* 6803 model *i*Syn731^18^. In order to characterize the wild-type phenotype, we utilized ^13^C MFA flux estimations^19^ under photosynthetic condition. Below is the step-by-step procedure that we followed:

#### **Step 1**

Identify the maximum biomass and limonene yields under photosynthetic condition.

Maximize *v*_*biomass*_ or *v*_*ls*_

Subject to

1 $$\sum _{j=1}^{m}{s}_{ij}{v}_{j}=0\forall {\rm{i}}\in 1,\ldots \ldots ,{\rm{n}}$$

2 $${a}_{j}{v}_{j}^{min}\le {v}_{j}\le {a}_{j}{v}_{j}^{max}\forall \,j\in 1,\ldots \ldots ,{\rm{m}}$$

3 $$0\le {v}_{Nutrients}\le {v}_{Nutrients}^{{\rm{\max }}}\forall \,{\rm{Nutrients}}\in {\rm{Light}},\text{Carbon}\,\text{source}({\rm{s}}),{\rm{Micro}}-{\rm{nutrients}}$$

#### **Step 2**

Characterize the wild-type phenotype

Maximize/Minimize ${v}_{j}\forall j\in$ reactions without experimental flux measurements

Subject to

1 $$\sum _{j=1}^{m}{s}_{ij}{v}_{j}=0\forall {\rm{i}}\in 1,\ldots \ldots ,{\rm{n}}$$

2 $${a}_{j}{v}_{j}^{min}\le {v}_{j}\le {a}_{j}{v}_{j}^{max}\forall j\in 1,\ldots \ldots ,{\rm{m}}$$

3 $$0\le {v}_{Nutrients}\le {v}_{Nutrients}^{{\rm{\max }}}\forall {\rm{Nutrients}}\in {\rm{Light}},{\rm{Carbon}}\,{\rm{source}}({\rm{s}}),{\rm{Micro}}-{\rm{nutrients}}$$

4 $${v}_{biomass}\ge {v}_{biomass}^{max}$$

#### **Step 3**

Characterize the limonene over-producing phenotype

Maximize/Minimizev_*j*_ ∀ j ∈ 1, ……,*m*

Subject to

1 $$\sum _{j=1}^{m}{s}_{ij}{v}_{j}=0\forall {\rm{i}}\in 1,\ldots \ldots ,{\rm{n}}$$

2 $${a}_{j}{v}_{j}^{min}\le {v}_{j}\le {a}_{j}{v}_{j}^{max}\forall j\in 1,\ldots \ldots ,{\rm{m}}$$

3 $$0\le {v}_{Nutrients}\le {v}_{Nutrients}^{{\rm{\max }}}\forall {\rm{Nutrients}}\in {\rm{Light}},{\rm{Carbon}}\,{\rm{source}}({\rm{s}}),{\rm{Micro}}-{\rm{nutrients}}$$

5 $${v}_{biomass}\ge 0.1{v}_{biomass}^{max}$$

6 $${v}_{ls}\ge 0.9{v}_{ls}^{max}$$

#### **Step 4**

Identify the MUST sets

In this step, fluxes ranging from step 2 and step 3 were compared to identify three different sets: reactions to be up-regulated (*MUST* ^*U*^), down-regulated (*MUST* ^*L*^), and deleted (*MUST* ^*X*^).

#### **Step 5**

Identify the minimal engineering interventions

Maximize *v*_*j*_

(**over** ***MUST*** **sets**)

Subject to

Minimize *v*_*j*_

(**over** ***MUST*** **sets**)

Subject to

1 $$\sum _{j=1}^{m}{s}_{ij}{v}_{j}=0\forall {\rm{i}}\in 1,\ldots \ldots ,{\rm{n}}$$

2 $${a}_{j}{v}_{j}^{min}\le {v}_{j}\le {a}_{j}{v}_{j}^{max}\,\forall \,j\,\in \,1,\ldots \ldots ,{\rm{m}}$$

3 $$0\le {v}_{Nutrients}\le {v}_{Nutrients}^{{\rm{\max }}}\forall {\rm{Nutrients}}\in {\rm{Light}},{\rm{Carbon}}\,{\rm{source}}({\rm{s}}),{\rm{Micro}}-{\rm{nutrients}}$$

5 $${v}_{biomass}\ge 0.1{v}_{biomass}^{max}$$

7 $${\rm{MUST}}\,{\rm{set}}\,{\rm{conditions}}$$

8 $${\sum }^{}\#of\,direct\,manipulations\le k$$

Here, *S*_*ij*_ is the stoichiometric coefficient of metabolite *i* in reaction *j* and *v*_*j*_ is the flux value of reaction *j*. Parameters *v*_*j*,*min*_ and *v*_*j*,*max*_ denote the minimum and maximum allowable fluxes for reaction *j*, respectively. *V*_*biomass*_ and *v*_*ls*_ represent biomass and limonene synthesis reactions under photosynthetic conditions, whereas *v* ^*max*^_*biomass*_ and *v* ^*max*^_*ls*_ represent the maximum theoretical yields of biomass and limonene under photosynthetic conditions. The minimal levels of biomass and the minimal target yield of limonene were set to be 10% of maximum biomass and 85% or 90% of maximum limonene yield, respectively. Finally, k represents the maximum number of interventions allowed.

### Pigment content analysis

Cell cultures (1 mL) were collected by centrifugation at 16,000 x *g* for 7 min, and the supernatants were removed. To extract pigments in *Synechocystis*, pre-cooled methanol (1 mL) was added to the pellets, and mixed thoroughly by pipetting and vortexing. Samples were incubated at 4 °C for 20 mins, and centrifuged at 16,000 x *g* for 7 min. The supernatants were removed for a spectroph-otometer analysis to quantify the concentrations of carotenoids and chlorophyll. The following equations were used to calculate the pigment content: $\text{chlorophyll}\,({\rm{\mu }}{\rm{g}}/{\rm{mL}})=(16.29\times {A}_{665})-(8.54\times {A}_{552})$ ^40^; $\text{carotenoids}\,({\rm{\mu }}g/\text{mL})=[(1000\times {A}_{470})-$ $(2.86\times Ch{l}_{a}[\mu g/{mL}])]/221$ ^41^.
